# Refining Left Bundle Branch Block Activation Criteria in CRT Candidates: A Noninvasive Electrocardiographic Imaging Study

**DOI:** 10.3390/diagnostics16111724

**Published:** 2026-06-03

**Authors:** Lev Malishevskii, Stepan Zubarev, Anastasia Bazhutina, Nikita Markov, Evgeny N. Mikhaylov, Tatiana Chumarnaya, Vera Stepanova, Viktoria Lebedeva, Olga Solovyova, Dmitry S. Lebedev

**Affiliations:** 1 Almazov National Medical Research Centre, 197341 Saint Petersburg, Russiae.mikhaylov@almazovcentre.ru (E.N.M.);; 2 Institute of Immunology and Physiology, Ural Branch of the Russian Academy of Sciences, 620078 Ekaterinburg, Russia; banas49@mail.ru (A.B.); ns.markov@urfu.ru (N.M.);; 3 Department of Mathematics, Mechanics and Computer Science, Ural Federal University, 620002 Ekaterinburg, Russia; 4 I.I. Mechnikov North-Western State Medical University, 191015 Saint Petersburg, Russia; veragrokhotova@mail.ru

**Keywords:** cardiac resynchronization therapy, left bundle branch block, noninvasive activation mapping, ECG imaging, endocardial mapping

## Abstract

**Background/Objectives:** Invasive mapping studies have shown that one-third of patients with an electrocardiographic pattern of LBBB have intact left bundle branch block conduction and therefore do not actually have a substrate for resynchronization. We aimed to evaluate the activation pattern among responders and non-responders to CRT in a relatively large patient cohort using noninvasive electrocardiographic imaging (ECGi). **Methods:** A cohort of 185 patients who underwent CRT implantation were enrolled. ECGi was performed at the same hospitalization during intrinsic rhythm. The earliest and the latest left ventricular (LV) activation sites were analyzed according to the 17-segment American Heart Association model. The echocardiographic response to CRT was defined as a reduction of ≥15% in LV end-systolic volume, and clinical response as an improvement of at least one New York Heart Association class at 12 months from the baseline. **Results:** The earliest activation in the middle inferoseptal LV segment (No.9) was significantly more prevalent among non-responders. A breakthrough in the remaining septal segments (No.2, 3, 8, or 14) was an independent predictor of both clinical (odds ratio (OR) 3.1 [95% confidence interval (CI): 1.2–8.1], *p* = 0.021) and echocardiographic (OR 5.1 [95% CI: 2.2–11.8], *p* < 0.001) responses to CRT. Other independent predictors of echocardiographic and clinical response to CRT in this study were LV pacing site in the lateral wall and QLV interval. **Conclusions:** The earliest endocardial activation site in segments 2, 3, 8, or 14 of the LV is an independent intrinsic activation marker associated with CRT response, complementary to intraoperative factors.

## 1. Introduction

Diagnosis of a complete left bundle branch block (LBBB) using the standard 12-lead electrocardiogram (ECG) had long been considered a resolved issue in the field of cardiology and interventional electrophysiology. However, in 1984, the first results of endocardial mapping in patients with LBBB revealed heterogeneous left ventricular (LV) activation sequences [1].

The inclusion of LBBB in clinical guidelines as one of the primary selection criteria for cardiac resynchronization therapy (CRT) in patients with chronic heart failure (CHF) has further increased interest in this field [2,3,4]. In 2004, Auricchio et al. demonstrated that approximately one-third of CRT candidates with LBBB on the standard 12-lead electrocardiogram (ECG) exhibited normal direction (left-to-right) and time (<20 ms) of transseptal activation during endocardial mapping [5]. The absence of LBBB, despite a similar ECG pattern, might clarify why a considerable number of patients do not respond to CRT [5,6,7,8,9,10].

To improve patient selection for CRT, numerous invasive electrophysiological studies have been conducted to investigate the ventricular activation pattern in LBBB and CHF. These studies have established several key characteristics of the LBBB activation pattern: ventricular activation originates from the endocardium of the right ventricle (RV); delayed transseptal conduction is recorded (>20 or >40 ms); the sole breakthrough into the LV endocardium is localized within the septum (especially in the middle and apical segments); and a U-shaped activation pattern of the LV is formed [5,6,7,8,9,10]. Due to their invasive nature, however, these studies included only small patient groups, leaving many aspects of ventricular activation in LBBB poorly understood.

Noninvasive activation mapping, or ECG imaging (ECGi), appears to address the limitations of invasive methods, enabling the assessment of electrophysiological processes on both the epicardial and endocardial surfaces of the heart’s ventricles. Recent evidence has shown that ECGi can aid in predicting response to CRT [11,12]. Notably, previous ECGi studies predominantly focused on assessing the epicardial surface of the heart and, therefore, could not assess the location of the earliest activation on the LV endocardium. However, some currently available ECGi systems may assess endocardial LV activation with relatively high accuracy [13,14].

We sought to analyze the ECGi-based activation sequences of the ventricular endocardium and epicardium in CRT responders vs. non-responders.

## 2. Materials and Methods

This single-center non-randomized study included a cohort of 185 patients with chronic heart failure and indications to BiV-CRT according to clinical guidelines relevant at the time of implantation. All enrolled participants signed an informed consent form. All patients underwent implantation of a BiV-CRT system between 2018 and 2022 at the Almazov National Medical Research Center. The study adhered to Good Clinical Practice guidelines and followed the principles outlined in the Declaration of Helsinki. The study protocol was approved by the Ethics Committee of the Almazov National Medical Research Center. Before CRT device implantation, a 12-lead ECG was recorded for all patients. LBBB was classified based on the ECG criteria from the American Heart Association (AHA) [2], Strauss et al. [6], the European Society of Cardiology (ESC) 2013 [4], and the ESC 2021 [3].

### 2.1. CRT Response Definitions

Echocardiographic response to CRT was defined as a ≥15% reduction in LV end-systolic volume (LV ESV) at 12 months from the baseline. Clinical response to CRT was defined as an improvement of at least one New York Heart Association (NYHA) class compared to baseline. NYHA class was assessed by a 6 min walk distance.

### 2.2. Electrocardiographic Imaging

All patients underwent ECGi using the system produced by Amycard LLC and EP Solutions SA (Yverdon-les-Bains, Switzerland) [14,15,16]. ECGi was performed after CRT implantation during the same hospitalization, following temporary stimulation inhibition for at least one minute to record the patient’s intrinsic rhythm. The chosen design permitted CT-based localization of the LV pacing site, measurement of the QLV interval, and recording of LV lead type and CRT programming parameters on the same anatomical model and within the same imaging session as the activation analysis. Because reverse electrical and structural remodeling induced by CRT develops over weeks to months rather than hours, the intrinsic activation sequence recorded after temporary inhibition of biventricular pacing reflects the patient’s underlying baseline conduction substrate. Throughout the manuscript, the activation pattern obtained in this manner is therefore referred to as an intrinsic activation marker.

**Figure 1:**
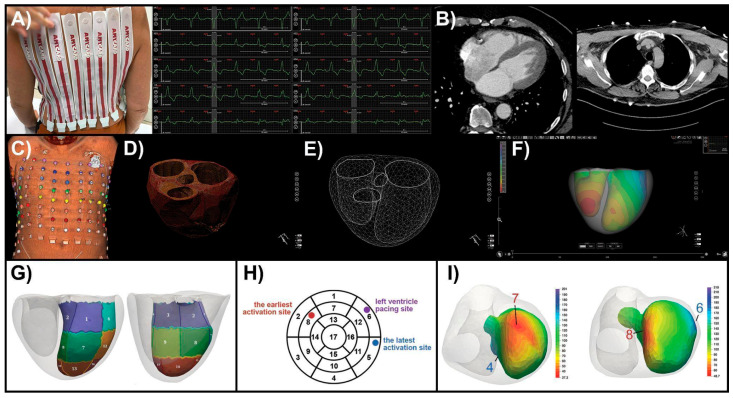
Workflow of the ECGi: (**A**) multichannel ECG; (**B**) computed tomography (torso and heart); (**C**) determining the spatial coordinates of the ECG electrodes on the torso model; (**D**) voxel model of the ventricles; (**E**) polygonal model of the ventricles; (**F**) polygonal model of the ventricles with automatically generated isochronal map; (**G**) automatic segmentation of the LV according to the AHA model; (**H**) annotation of the LV pacing site, the earliest and the latest activation sites in the corresponding LV segments; (**I**) examples of the resulting LV activation maps with LV segment numbers of the earliest and the latest activation sites.

Briefly, the ECGi workflow is presented in Figure 1.

The approach used to solve the inverse problem of ECG was the equivalent single-layer (ESL) algorithm described earlier in [16]. Validation of the Amycard electrophysiology system for noninvasive epi- and endocardial mapping has been reported previously [14,15,16]. Analysis of the endocardial surface allows inclusion of the interventricular septum in the measurements [15].

All personalized LV endocardial models were divided into 17 segments according to the 17-segment LV model proposed by the AHA [17]. This segmentation was performed using a previously validated automatic algorithm based on the patient’s CT-derived anatomy [18] and did not involve operator-dependent visual assignment. Segment boundaries were anchored on reproducible CT-derived anatomical landmarks (LV apex, basal mitral plane, and anterior and posterior right-ventricular insertion points). The algorithm subsequently identifies the earliest and latest activation sites on both the LV endocardium and epicardium, returning the segment number of each site together with its activation time. In cases where multiple early or late sites occurred across different segments, the point and segment with the most pronounced value were selected.

The median total LV endocardial area in the present cohort was 181.2 cm^2^ [151.6; 227.0]; the LV endocardial septum (segments 2, 3, 8, 9, and 14 combined) had a median area of 67.6 cm^2^ [56.3; 78.7], and individual septal segment areas ranged from a median of 12.5 cm^2^ to 14.3 cm^2^. At this anatomical scale, characteristic of the dilated ventricles of CRT candidates, each septal segment constitutes a well-defined regional territory rather than a micro-localization. To further confirm that breakthrough points are spatially well separated between adjacent AHA segments rather than scattered along their borders, their positions were analyzed in the normalized space of Universal Ventricular Coordinates (UVC). Since breakthrough points lie on the LV endocardium, the transmural (ρ = 0) and LV/RV (ν = 1) coordinates were fixed, and the apicobasal coordinate z (normalized to [0, 1] per patient) and the angular coordinate θ (normalized to [−1, 1] by division by π) were used. One-way MANOVA in this two-dimensional space showed significant discrimination between segments (Wilks’ λ = 0.168, F = 67.8, *p* < 0.001), with θ as the dominant discriminating axis (F = 118.8). Silhouette analysis assigned 86.7% of breakthrough points to their respective segments with positive spatial confidence (mean silhouette coefficient = 0.44). Within the septal segments specifically addressed by the activation criterion, silhouette values were also consistent with reliable segment-level assignment and did not differ between responders and non-responders.

For each isochronal map, temporal characteristics of ventricular activation were automatically calculated. The total activation time (TAT) of the heart ventricles was determined by computing the time interval between the earliest and latest activation time among cardiac mesh points. The activation times of the LV (LVAT) and RV (RVAT) were calculated as the differences between the time of the latest activation and the time of the earliest activation within the LV and RV, respectively. The transseptal conduction time was measured from the onset of the QRS complex to the time of the earliest activation site on the LV endocardium [5]. Ventricular electrical uncoupling (VEU) was defined as the difference between the mean LV and RV activation times [11].

### 2.3. Left Ventricular Pacing Site, Interlead Distance, and QLV Interval Analysis

The LV pacing site (LVPS) was manually identified on cardiac CT images using the software’s fluoroscopy visualization mode, coupled with data on LV lead position, the number of active poles, and their positions. The LVPS was then annotated on the ventricular mesh model. An integer from 1 to 17 was assigned to the LVPS, corresponding to the segment number in the 17-segment AHA model.

Interlead distance was measured as the Euclidean distance between active stimulation poles of left (LVPS) and right leads on a three-dimensional model of the ventricles based on CT data. A noninvasive QLV measurement was performed using EP Solutions software, as previously described by Varma et al. [19]. The measurement was defined as the time interval from the first deflection on a surface electrocardiogram to local intrinsic activation at the LVPS.

### 2.4. Follow-Up

The follow-up period was limited to 12 months after implantation. Patients were examined during their hospitalization for CRT implantation and subsequently at the 3rd and 12th month post-implantation. The follow-up examination included clinical assessment, echocardiographic evaluation, 6 min walk distance, and CRT programming. Background medical therapy was reviewed at each follow-up visit and adjusted as clinically required, in accordance with the institutional standard for heart-failure management. The CRT device underwent optimization during the index hospitalization. Re-optimization was carried out at 3 months, at 12 months, and then annually thereafter. Unscheduled visits occurred at the patient’s request upon worsening of clinical symptoms, to modify CRT programming parameters and adjust drug therapy. All patients were followed up by a team of physicians, including a heart failure specialist, electrophysiologist, and general cardiologist.

### 2.5. Statistical Analysis

The statistical analysis was performed using IBM SPSS version 28.0.1. Discrete variables are expressed as counts (percentages). Quantitative data are presented as mean ± standard deviation (M ± SD) for normally distributed data, assessed by the Kolmogorov–Smirnov test with the Lilliefors correction. For non-normally distributed data, the median with interquartile range (Me [Q1; Q3]) is used. In tables, quantitative data are presented as Me [Q1; Q3]. Variables were compared between the two outcomes (non-responder vs. responder) in two definitions (LV ESV and NYHA): either Student’s *t*-test or the Mann–Whitney test was used to analyze quantitative data, depending on the distribution; qualitative variables were analyzed using Pearson’s chi-square test. For the factors deemed significant, we created univariable logistic regression models to assess their predictive association with CRT outcomes. Consequently, factors found statistically significant were selected for inclusion in the multivariable logistic regression models independently for two CRT response definitions. Odds ratios (ORs) with 95% confidence intervals (CIs) and *p*-values were calculated for univariable and multivariable analysis.

To compare the predictive performance of the proposed activation criterion and of the multivariable logistic regression model with the standard ECG criteria for LBBB, we performed a ROC analysis. The area under the curve (AUC) with 95% confidence interval was calculated for each predictor and for both response definitions. The significance of each AUC against the no-discrimination value of 0.5 was assessed by the Mann–Whitney U test. Pairwise comparisons of AUC values between predictors were performed using the DeLong test. In addition, Spearman’s correlation coefficient was used to assess the relationship between segments of the latest activation site and LVPS. Statistical significance α was set at *p* < 0.05, with Bonferroni correction applied when comparing three groups (*p* < 0.017).

## 3. Results

Among 185 included patients, 73% were males, and the median age at implantation was 65 [59; 70] years. At 12 months of follow-up, 126 (68.1%) patients demonstrated the echocardiographic response, while 135 (73%) patients showed the clinical response to CRT. The patients’ clinical characteristics at baseline are presented in Table 1.

The echocardiographic and clinical response groups were comparable regarding main clinical and functional characteristics at baseline. The use of quadripolar LV leads, automatic CRT optimization algorithms, and LVPS localization in the lateral wall were significantly more prevalent among responders according to the echocardiographic definition (Table 1).

Localization of the baseline earliest and the latest activation sites of the left ventricle.

**Figure 2:**
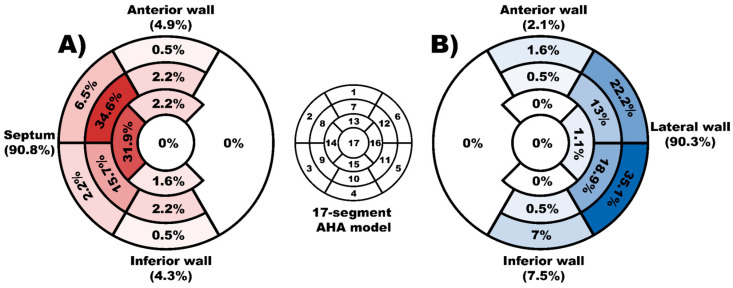
Localization of the earliest (**A**) and the latest (**B**) activation sites in the LV (*n* = 185).

In most cases (90.8%), the earliest activation site was observed within the septal segments of the LV endocardium (Figure 2A). The latest activation site was registered exclusively on the epicardial surface, predominantly on the lateral wall (Figure 2B).

A significant association was observed between the earliest endocardial activation site in the LV walls (anterior wall, septum, inferior wall) and the echocardiographic response to CRT (Table 2). When two groups were compared, a breakthrough into the septum was significantly more prevalent among echocardiographic responders (95.2% vs. 81.4%, *p* = 0.005).

There was a significant association between the localization of the latest activation site in LV walls (anterior, lateral, or inferior) and the clinical response to CRT (Table 2). When comparing two groups, it was found that the localization of the latest site on the lateral wall was significantly more prevalent among clinical responders (94.1% vs. 80%, *p* = 0.009).

### 3.1. Subanalysis of the Earliest and Latest LV Segments Associated with Response to CRT

**Figure 3:**
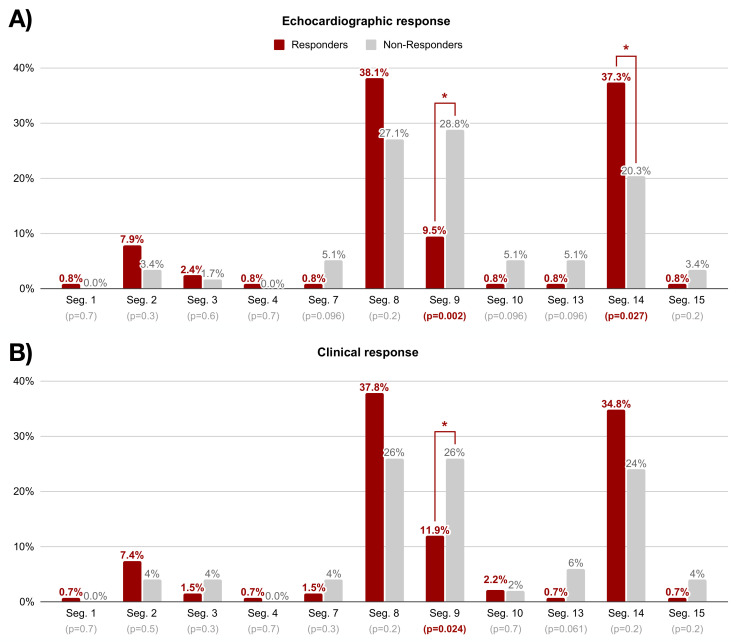
Localization of the earliest activation site in LV endocardial segments among (**A**) echocardiographic and (**B**) clinical responders vs. non-responders. * *p* < 0.05.

Although responders demonstrated higher rates of earliest septal activation, there was no significant correlation between septal segments and response when the segments were evaluated independently (Figure 3). Only early activation in segment 14 was significantly associated with echocardiographic CRT response.

The lack of a significant association with response may indicate that early activation in one segment cannot serve as a selection criterion, and perhaps they need to be grouped. Previous invasive mapping studies have shown that early activation in any septal segment indicates an absence of preserved conduction along LBB fibers [1,8,9]. However, in our study, early activation in the mid-inferior septal segment (segment 9, *n* = 29) was significantly more prevalent in CRT non-responders according to both definitions.

Thus, early activation in the septum was associated with a response to CRT. However, when the individual segments were analyzed, early activation in segment 9 (*n* = 29) was significantly more common among non-responders. Considering a lower likelihood of CRT response when a breakthrough occurred in segment 9, we hypothesized that the remaining segments of the septum may have a significant association with response to CRT. A breakthrough in the anterior or posterior walls of the LV with preserved conduction indicates the absence of LBBB, despite the ECG pattern. This is associated with non-response to CRT. Given our results, early activation in segment 9 may also need to be considered in this group of patients.

**Figure 4:**
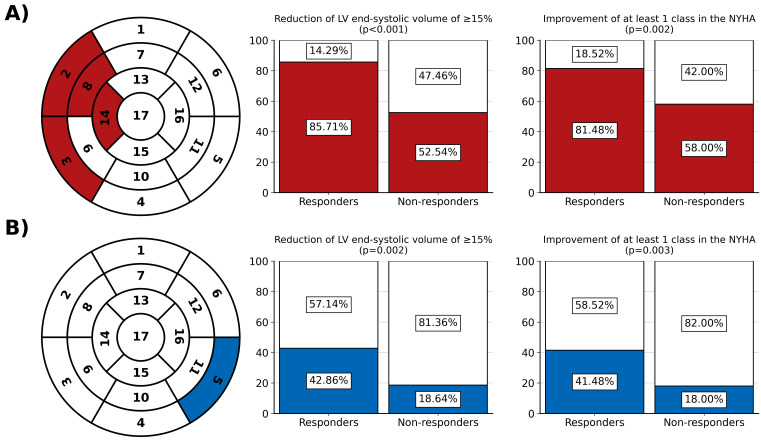
The association between the response to CRT and (**A**) the earliest activation in segments 2, 3, 8, or 14; (**B**) the latest activation in segment 5.

Subsequently, patients with the breakthrough localized in segments 2, 3, 8, or 14 (*n* = 139) had a significant association with both echocardiographic and clinical response to CRT, compared with early activation in the (*n* = 46): anterior (segments 1, 7, or 13), posterior (segments 4, 10, or 15) walls, or segment 9 of the LV (Figure 4A).

**Figure 5:**
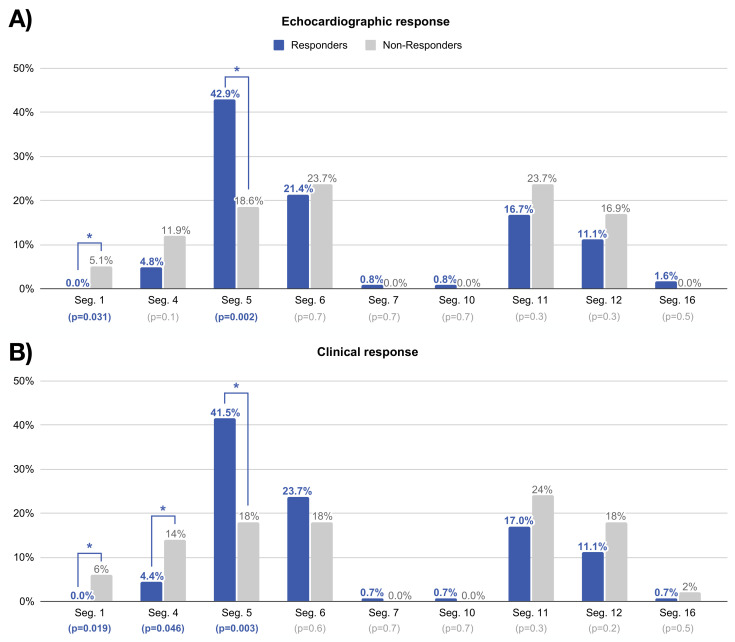
Localization of the latest activation site in LV epicardial segments among (**A**) echocardiographic and (**B**) clinical responders vs. non-responders. * *p* < 0.05.

The latest activation site in segment 5 was significantly more prevalent among responders compared to non-responders (Figure 4B and Figure 5).

In contrast, basal segments of the anterior (No.1) or posterior (No.4) walls of the LV were associated with a negative response to CRT (Figure 5). The remaining segments showed no significant association with the CRT response (Figure 5).

No significant correlation was observed between CRT response and the localization of the latest activation site in other LV segments of the lateral wall. No statistically significant correlation was identified between the segments of the LVPS and the latest activation site (r2 = −0.016, *p* = 0.83).

### 3.2. Ventricular Activation Time

The median TAT for all patients was 176 [165; 194] ms. Nearly all patients (97.3%) had ventricular activation that originated on the RV endocardium and subsequently progressed to the LV endocardium with an average transseptal delay of 38.4 ± 17.4 ms. The mean LVAT was 135.4 ± 27.1 ms, the mean RVAT was 115.9 ± 21.4 ms, and the mean VEU was 48.7 ± 11.4 ms. No significant differences were detected in ventricular activation time between the response groups.

Patients with the breakthrough observed in segments 2, 3, 8, or 14 vs. other patients (breakthrough in segment 9, anterior or inferior walls) exhibited significantly longer transseptal conduction time (39.9 ± 16.7 vs. 33.8 ± 18.6, *p* = 0.04) and VEU (50.2 ± 10.5 vs. 44.3 ± 13.1, *p* = 0.002) compared to remaining patients. The latest activation in segment 5 of the LV was not associated with any of the analyzed ventricular activation times.

### 3.3. LV Pacing Site

**Figure 6:**
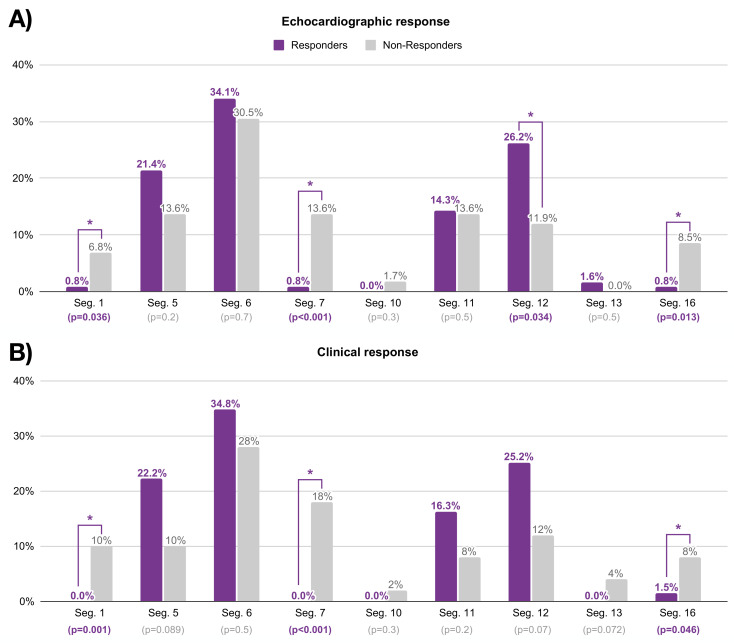
Localization of the left ventricle pacing site in LV epicardial segments among (**A**) echocardiographic and (**B**) clinical responders vs. non-responders. * *p* < 0.05.

A subanalysis of LVPS location revealed significant differences between response groups based on electrode placement in specific segments (Figure 6): basal anterior (No.1), mid anterior (No.7), mid anterolateral (No.12), and apical lateral (No.16). LVPS on the anterior wall (segments No.1 and No.7) was significantly more prevalent among both echocardiographic and clinical non-responders, while early activation in segment 12 was associated with echocardiographic response (Figure 6).

Although LVPS in the lateral wall generally correlated with a positive CRT response (Table 1), placement in the apical lateral segment (No.16) was significantly more frequent among non-responders by both echocardiographic and clinical definitions (Figure 6). Therefore, patients with localization in the remaining lateral wall LV segments (No.5, 6, 11, or 12) were grouped together for further analysis. LVPS in these segments was significantly associated with both echocardiographic (96% vs. 69.5%, *p* < 0.001) and clinical (98.5% vs. 58%, *p* < 0.001) response to CRT.

### 3.4. Univariable Analysis of Significant Factors

To further analyze the predictive association of the described factors with CRT response, we performed univariable analysis using logistic regression. Table 3 shows the results in terms of calculated odds ratios in two CRT response definitions for each factor.

### 3.5. Multivariable Analysis

A multivariable analysis was conducted to examine the factors that were significantly associated with the response to CRT in the univariable analysis.

The earliest activation in segments 2, 3, 8, or 14; QLV interval; and LVPS in the 5, 6, 11, or 12 segments, were identified as independent predictors of both clinical and echocardiographic response, irrespective of other factors (Table 4).

### 3.6. ROC Analysis

**Figure 7:**
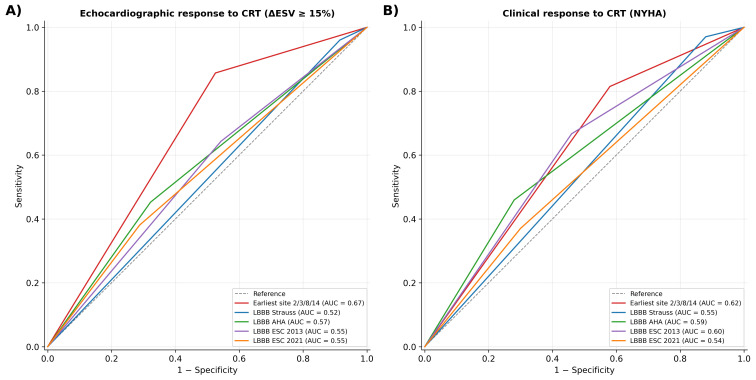
ROC curves for prediction of (**A**) echocardiographic (reduction in LV ESV by ≥15%) and (**B**) clinical (improvement of at least one NYHA class) response to CRT, comparing the proposed activation criterion (earliest activation in segments 2, 3, 8, or 14) with the AHA [2], Strauss [6], ESC 2013 [4], and ESC 2021 [3] ECG criteria for LBBB.

Among the three independent predictors identified in the multivariable analysis, only the earliest activation in segments 2, 3, 8, or 14 is preoperatively available; LVPS and QLV interval are intraoperative parameters. To assess the discriminative performance of the proposed activation criterion against the standard ECG criteria for LBBB, we performed a ROC analysis for both response definitions (Figure 7).

For the echocardiographic response, the activation criterion yielded an AUC of 0.67 [95% CI: 0.60–0.74], *p* < 0.001. None of the ECG criteria for LBBB reached statistical significance (LBBB AHA AUC 0.57 [0.49–0.64], *p* = 0.086; LBBB ESC 2013 AUC 0.55 [0.47–0.63], *p* = 0.199; LBBB ESC 2021 AUC 0.55 [0.47–0.62], *p* = 0.208; LBBB Strauss AUC 0.52 [0.48–0.56], *p* = 0.266). The DeLong test showed that the AUC of the activation criterion was significantly higher than that of the LBBB Strauss (ΔAUC 0.14, *p* < 0.001), LBBB ESC 2013 (ΔAUC 0.12, *p* = 0.019), and LBBB ESC 2021 (ΔAUC 0.12, *p* = 0.015) criteria; the comparison with LBBB AHA was borderline (ΔAUC 0.10, *p* = 0.051).

For the clinical response, the AUC of the activation criterion was 0.62 [0.54–0.69], *p* = 0.003. Two of the four ECG criteria for LBBB also reached significance: LBBB ESC 2013 (AUC 0.60 [0.52–0.68], *p* = 0.012), LBBB AHA (AUC 0.59 [0.51–0.67], *p* = 0.020), and LBBB Strauss (AUC 0.55 [0.50–0.59], *p* = 0.063, borderline). LBBB ESC 2021 was not significant (AUC 0.54 [0.46–0.61], *p* = 0.365). Pairwise DeLong comparisons did not reveal significant differences between the activation criterion and any of the ECG criteria for the clinical response (all *p* > 0.05).

**Figure 8:**
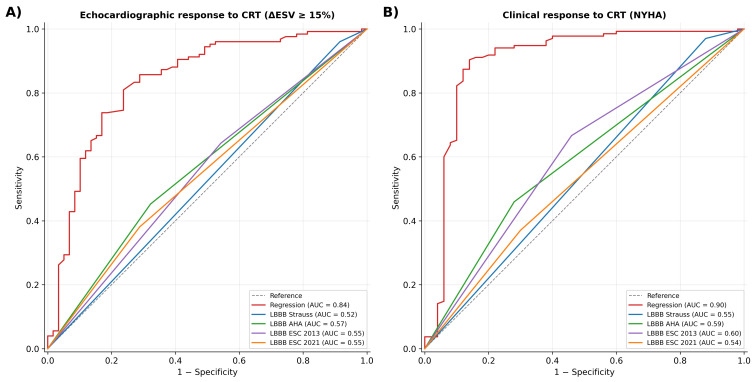
ROC curves for prediction of (**A**) echocardiographic (reduction in LV ESV by ≥15%) and (**B**) clinical (improvement of at least one NYHA class) response to CRT, comparing the multivariable logistic regression model with the AHA, Strauss, ESC 2013, and ESC 2021 ECG criteria for LBBB.

ROC analysis was also performed for the multivariable logistic regression model itself, using its linear predictor as the score (Figure 8). The combined model, integrating the activation criterion (segments 2, 3, 8, or 14), LVPS (segments 5, 6, 11, or 12), and QLV interval, reached an AUC of 0.84 [0.77–0.90], *p* < 0.001 for the echocardiographic and 0.90 [0.84–0.97], *p* < 0.001 for the clinical response. The model was significantly superior to each of the four ECG criteria for both response definitions (DeLong *p* < 0.001 for all pairwise comparisons).

## 4. Discussion

The major finding of our study is that during intrinsic rhythm, the earliest activation in the middle inferoseptal segment (9) was significantly more prevalent among clinical and echocardiographic non-responders. The earliest endocardial activation in the remaining segments of the septum (2, 3, 8, or 14) was independently associated with clinical and echocardiographic response to CRT at 12-month follow-up. This association remained significant irrespective of the presence of ECG criteria for LBBB, the type of LV lead, LVPS localization, QLV interval, use of automatic CRT optimization algorithm, or the latest activation site.

Current ESC and AHA guidelines on cardiac pacing and CRT [2,3,4] base patient selection on QRS morphology (LBBB by surface ECG) and QRS duration and do not incorporate substrate-level information from activation mapping. Despite successive refinement of these QRS-based criteria, approximately one-third of guideline-eligible patients fail to respond to CRT. This pattern was reproduced in the present cohort, in which 32% did not respond by the echocardiographic definition and 27% by the clinical definition, despite 94.6% meeting the Strauss LBBB criteria, 70.8% meeting the ESC 2013 criteria, and a median QRS duration of 176 [165; 194] ms. This residual non-response is consistent with the invasive observation [5] that approximately one-third of patients with an LBBB ECG pattern retain preserved transseptal conduction and therefore lack the electrophysiological substrate amenable to resynchronization.

Invasive activation mapping studies have shown that in LBBB, endocardial breakthrough in the LV during intrinsic rhythm occurs in the septum [1,5,8,9,10]. When an endocardial breakthrough appears in the anterior or posterior LV walls despite the presence of LBBB on an ECG, this suggests preserved conduction along the anterior or posterior fascicles of the left bundle branch [1,8,9]. In such cases, the wide QRS complex stems from nonspecific intraventricular conduction delay. Consequently, septal breakthrough is a widely accepted criterion for distinguishing true and false LBBB with the same ECG pattern. In our study, the earliest activation in the middle inferoseptal segment (9) unexpectedly showed a significantly higher prevalence among clinical and echocardiographic non-responders. After excluding segment 9, a breakthrough in the septum (segments 2, 3, 8, or 14) was found to be an independent predictor of clinical (OR 3.1 [95% CI: 1.2–8.1], *p* = 0.021) and echocardiographic (OR 5.14 [95% CI: 2.2–11.8], *p* < 0.001) response to CRT irrespective of the presence of ECG criteria for LBBB, the type of the LV lead, LVPS, QLV, or the latest activation site.

These results are consistent with the findings of invasive activation mapping studies. In 2003, Rodriguez et al. analyzed 12 patients with preliminary detailed mapping of the ventricular conduction system using three-dimensional non-fluoroscopic navigation systems [8]. They observed that in normal hearts, the earliest LV activation was recorded in the anterobasal LV (in the anterior fascicle) and in the mid septum (in the posterior fascicle). Rodriguez et al. found that some patients with wide QRS and LBBB morphology also demonstrated activation “in or close to the posterior fascicle” in the mid-posteroseptal region, which suggests preserved conduction along conduction system fibers [8]. Similarly, in our study, breakthrough in the mid-inferoseptal segment (segment 9) was more common in non-responders. Based on these observations, we hypothesize that a breakthrough in segment 9 is associated with intact conduction along the posterior branch of the LBB and serves, along with breakthroughs in the anterior and posterior LV walls, as an activation criterion for false LBBB. Within-septum classification uncertainty does not affect the proposed criterion. The nearest neighbors of segment 9 in UVC space are segments 3 and 8, both of which belong to the responder group {2, 3, 8, 9, 14}. A misclassified breakthrough would therefore stay within this group and not cross the criterion boundary.

Beyond its clinical implications, the present work contributes to the fundamental characterization of ventricular activation in true versus false LBBB. Direct evidence in this area has so far been confined to small invasive series [5,8]. The present study cannot independently verify this mechanism but provides large-cohort, noninvasive evidence that converges with the prior invasive observations of Rodriguez et al. [8]. Direct invasive conduction-system mapping would be needed to confirm the underlying substrate.

Prior noninvasive activation mapping studies have primarily focused on the epicardial LV activation sequence [11,12]. Ploux et al. detailed the sequence of epicardial activation patterns in LBBB and IVCD. Parreira et al. showed that it is crucial to prioritize the localization of the latest activation site in relation to the LVPS to achieve effective resynchronization. We performed a qualitative analysis of the latest site and LVPS using a 17-segment AHA model. When comparing the different walls of the LV, the localization of the latest site on the lateral side was found to be significantly more common in clinical responders. Detailed analysis revealed that the latest site in the basal inferolateral segment (#5) was significantly more prevalent in echocardiographic and clinical responders compared to non-responders. However, the latest activation site was not an independent predictor of response when other factors were considered. On the other hand, LVPS in the basal or mid-segments of the lateral wall was identified as the strongest independent predictor of both clinical (OR 21.2 [95% CI: 4.3–105], *p* < 0.001) and echocardiographic (OR 4.42 [95% CI: 1.4–14.3], *p* = 0.025) response to CRT.

In addition to the LVPS, the electrical delay from the onset of the native QRS complex to the local LV electrogram (QLV interval) indicates the electrical position of the LV lead and can enhance CRT efficacy [19]. Our study also found that a longer QLV interval showed significant differences in multivariable analysis for both clinical (OR 1.08 [95% CI: 1.04–1.1], *p* < 0.001) and echocardiographic (OR 1.04 [95% CI: 1.015–1.066], *p* < 0.001) response.

Despite the importance of intraoperative predictors like LVPS and QLV interval, a significant advantage of the activation criteria is their availability at the preoperative stage. These criteria are valuable for selecting patients not only for biventricular pacing but also for cardiac conduction system pacing, where QLV interval or LVPS are not applicable.

Based on the study design, all enrolled patients met the contemporary clinical guideline indications for CRT, including a prolonged QRS, with a median QRS duration of 176 [165; 194] ms and 94.6% of patients meeting the Strauss LBBB criteria. Within this CRT-eligible population, QRS duration cannot further discriminate responders from non-responders, since its discriminative power has been exhausted at the inclusion stage.

The four ECG definitions classified the present cohort very differently: AHA 53.5%, Strauss 94.6%, ESC 2013 70.8%, and ESC 2021 28.6%. Such inter-criterion discordance is well documented. In a previous comparison of nine LBBB definitions used in guidelines and major CRT trials, agreement between Strauss and AHA/ESC criteria was only minimal (κ 0.24–0.34) [20]. Van Stipdonk et al., in a multicenter cohort, reported that only 13.8% of patients met all four contemporary LBBB definitions simultaneously, with pairwise κ ranging from 0.09 to 0.92 [21]. Caputo et al. demonstrated that the choice of LBBB definition substantially influences the observed CRT response [22]. The ESC 2021 criteria have been criticized for being overly restrictive: applying them in 1202 CRT patients reduced the proportion of LBBB diagnoses from 80.9% (ESC 2013) to 31.6%, and, unlike the 2013 definition, the 2021 definition no longer separated responders from non-responders by either echocardiographic or clinical outcomes [23]. In an unequivocal LBBB cohort, only 12% of patients and only 19% of CRT super-responders met the ESC 2021 criteria [24]. More than half of patients classified as non-LBBB by the ESC 2021 criteria nevertheless exhibited septal flash or apical rocking on echocardiography, with no difference in LV reverse remodeling between LBBB and non-LBBB groups under the new criteria [25].

Comparison of the proposed activation criterion with the ECG criteria for LBBB confirmed its higher discriminative ability for the echocardiographic response. The earliest activation in segments 2, 3, 8, or 14 (AUC 0.67) was the only preoperative predictor reaching statistical significance, and its AUC was significantly higher than that of the Strauss, ESC 2013, and ESC 2021 ECG criteria. For the clinical response, the activation criterion (AUC 0.62) and three of the four ECG criteria were also significant, but the AUC values of all single predictors remained moderate (0.54–0.62) and the differences between them were not significant. This is consistent with the very high prevalence of LBBB by ECG in the cohort (94.6% by the Strauss criteria), where ECG morphology criteria approach the limit of their discriminative capacity.

When the activation criterion was combined with the intraoperative predictors (LVPS and QLV interval) within the multivariable model, the AUC reached 0.84 for the echocardiographic and 0.90 for the clinical response, both significantly higher than any of the ECG criteria. These results support the view that the response to CRT depends on both substrate-related (earliest activation site) and delivery-related (LVPS, QLV) factors and that no single ECG-based criterion can fully discriminate responders from non-responders in a CRT-eligible population.

Based on the results of the current study and the findings of Rodriguez et al. [8], a revision of the activation criterion for LBBB is proposed from “the earliest activation within the septum” to “the earliest activation within the septum, excluding the mid-inferoseptal segment”.

### Limitations

The present study has several limitations. First, endocardial mapping had been previously validated only for zones of early excitation during pacing and ectopic excitations [14,15]. Even though our results are in line with invasive mapping studies, further research is needed for extended validation of biventricular endocardial activation.

Second, the study population was highly selected, with a QRS duration of 176 [165; 194] ms and the presence of Strauss criteria on the ECG in 95% of patients. However, despite the high median QRS width and percentage of patients with LBBB by the Strauss criterion, there was still a 30% non-response to CRT.

Third, hard clinical endpoints were not assessed in the present analysis. The conclusions are therefore based on the surrogate echocardiographic and clinical endpoints at 12 months, and prospective validation of the proposed activation criterion against hard outcomes is warranted in a follow-up study of the same cohort.

Fourth, ECGi was acquired during the same hospitalization as CRT implantation, after temporary inhibition of biventricular pacing, rather than strictly before the procedure. Although no measurable reverse remodeling could plausibly occur within this interval and the intrinsic-rhythm recording reflects the underlying baseline conduction substrate (Section 2.2), prospective validation in a strictly preoperative cohort remains warranted.

Fifth, segmentation pipelines are not yet fully harmonized across ECGi vendors. Although the AHA 17-segment model itself is a vendor-independent anatomical reference, external validation of the proposed criterion across different ECGi platforms is needed before broader clinical deployment.

Sixth, the automatic AHA-segmentation algorithm employed in the present work [18] was originally validated against expert manual segmentation on the LV epicardial surface, and a dedicated quantitative assessment of segment-level agreement on the LV endocardium has not yet been published. Although the UVC-space analysis reported in Section 2.2 confirmed statistically significant separation between segments 3, 8 and 9 (MANOVA *p* < 0.001; mean silhouette ≥ 0.37 in each segment), the spatial separation between adjacent septal segments is small in absolute terms, and local classification uncertainty between adjacent septal segments cannot be entirely excluded. Both the endocardial validation of the segmentation algorithm and the assessment of segment-level spatial reproducibility are the subject of a forthcoming methodological study by our group, and the results will be referenced when available.

Seventh, the proposed mechanism, namely preserved conduction along the posterior fascicle of the left bundle branch, was not directly verified by invasive conduction-system mapping. Dedicated multimodal studies are needed to confirm the underlying substrate.

## 5. Conclusions

This electrocardiographic imaging study demonstrates that earliest left ventricular endocardial activation within septal segments 2, 3, 8, or 14, but not the mid-inferoseptal segment, is independently associated with cardiac resynchronization therapy response at 12 months. This finding challenges the current understanding that any slow septal breakthrough indicates a left bundle branch block. It provides a refined activation criterion for improved patient selection. Prospective validation in a strictly preoperative cohort and against hard clinical endpoints is warranted.

## Figures and Tables

**Table 1:** Clinical Characteristics at Baseline.

	All Patients (*n* = 185)	Reduction * in LV ESV by ≥15%			Improvement * of at Least One NYHA Class		
		Responders (*n* = 126)	Non-Responders (*n* = 59)	*p*	Responders (*n* = 135)	Non-Responders (*n* = 50)	*p*
Age, years	65 [59; 70]	65 [59; 70]	65 [59; 69]	0.6	65 [59; 70]	65 [60; 69]	0.7
Male gender, *n* (%)	134 (72.4%)	89 (70.6%)	45 (76.3%)	0.5	93 (68.9%)	41 (82%)	0.096
BMI, kg/m^2^	27.5 [25; 31]	27.6 [26; 30]	27.1 [25; 31]	0.5	27.7 [25; 30]	27.1 [25; 31]	0.5
NYHA III–IV functional class, *n* (%)	142 (76.8%)	95 (75.4%)	47 (79.7%)	0.6	102 (75.6%)	40 (80%)	0.6
Ischemic cardiomyopathy, *n* (%)	91 (49.2%)	59 (46.8%)	32 (54.2%)	0.4	64 (47.4%)	27 (54%)	0.5
Arterial hypertension, *n* (%)	159 (85.9%)	106 (84.1%)	52 (89.8%)	0.3	113 (83.7%)	47 (94%)	0.07
Anemia, *n* (%)	28 (15.1%)	21 (16.7%)	7 (11.9%)	0.4	22 (16.3%)	5 (10%)	0.3
Thyroid disease, *n* (%)	30 (16.2%)	21 (16.7%)	9 (15.3%)	0.8	21 (15.6%)	11 (22%)	0.3
Chronic kidney disease, *n* (%)	40 (21.6%)	27 (21.4%)	13 (22%)	0.9	27 (20%)	13 (26%)	0.4
Sinus rhythm, *n* (%)	167 (90.3%)	116 (92.1%)	51 (86.4%)	0.3	123 (91.1%)	44 (88%)	0.6
QRS, ms	176 [165; 194]	176.5 [164; 193]	175 [166; 196]	0.7	178 [166; 194]	174 [164; 195]	0.4
LV EF, %	27 [23; 31]	27.5 [24; 31]	26 [23; 32]	0.7	27 [23; 31]	27 [24; 32]	0.4
LV EDV, mL	240 [193; 317]	237.5 [187; 304]	262 [202; 323]	0.078	240 [189; 306]	252.5 [198; 326]	0.2
LV ESV, mL	180 [134; 233]	171.5 [129; 226]	190 [137; 257]	0.1	173 [130; 230]	183.5 [135; 261]	0.3
Beta-blocker, *n* (%)	183 (98.9%)	124 (98.4%)	59 (100%)	0.7	133 (98.5%)	50 (100%)	0.8
ACE inhibitor or ARB, *n* (%)	183 (98.9%)	126 (100%)	57 (96.6%)	0.3	133 (98.5%)	50 (100%)	0.8
Aspirin, *n* (%)	105 (56.8%)	73 (57.9%)	32 (54.2%)	0.5	76 (56.3%)	29 (58%)	0.8
Diuretics, *n* (%)	176 (95.1%)	117 (92.9%)	59 (100%)	0.06	126 (93.3%)	50 (100%)	0.1
Statins, *n* (%)	133 (71.9%)	87 (69.0%)	46 (78.0%)	0.2	96 (71.1%)	37 (74.0%)	0.7
Nitrates, *n* (%)	11 (5.9%)	7 (5.6%)	4 (6.8%)	0.8	9 (6.7%)	3 (6.0%)	1.0
LBBB AHA, *n* (%)	99 (53.5%)	57 (45.2%)	19 (32.2%)	0.1	**62 (45.9%)**	**14 (28%)**	**0.03**
LBBB Strauss, *n* (%)	175 (94.6%)	121 (96%)	54 (91.5%)	0.3	**131 (97%)**	**44 (88%)**	**0.025**
LBBB ESC 2013, *n* (%)	131 (70.8%)	81 (64.3%)	32 (54.2%)	0.2	**90 (66.7%)**	**23 (46%)**	**0.017**
LBBB ESC 2021, *n* (%)	53 (28.6%)	48 (38.1%)	17 (28.8%)	0.3	50 (37%)	15 (30%)	0.4
Use of automatic CRT optimization algorithm, *n* (%)	68 (38.8%)	**53 (42.1%)**	**15 (25.4%)**	**0.034**	53 (39.3%)	15 (30%)	0.3
Quadripolar LV lead, *n* (%)	59 (31.9%)	**47 (37.3%)**	**12 (20.3%)**	**0.027**	47 (34.8%)	12 (24%)	0.2
QLV interval	86 [76.5; 96.5]	**90 [82; 102.5]**	**76 [65; 86]**	**<0.001**	**90 [82; 103]**	**72 [60; 80]**	**<0.001**
LVPS in the lateral wall, *n* (%)	168 (90.8%)	**122 (96.8%)**	**46 (78%)**	**<0.001**	**135 (100%)**	**33 (66%)**	**<0.001**
LVPS in basal segments, *n* (%)	101 (54.6%)	71 (56.3%)	30 (50.8%)	0.5	77 (57%)	24 (48%)	0.3
Interlead distance, mm	90 [80; 102]	90 [81; 101]	89 [76; 102]	0.7	91 [81; 102]	86 [71; 100]	0.1

Quantitative data are presented as median with interquartile range (Me [Q1; Q3]). LV = left ventricle; ESV = end-systolic volume; NYHA = New York Heart Association; BMI = body mass index; EDV = end-diastolic volume; EF = ejection fraction; LBBB—left bundle branch block; LVPS—left ventricle pacing site; * At 12 months of follow-up.

**Table 2:** Localization of the earliest and the latest activation sites of the left ventricle (*n* = 185).

	Reduction * of LV ESV by ≥15%			Improvement * of at Least One NYHA Class		
	Responders (*n* = 126)	Non-Responders (*n* = 59)	*p*	Responders (*n* = 135)	Non-Responders (*n* = 50)	*p*
The earliest activation site						
Anterior wall (*n* = 9)	3 (2.4%)	6 (10.2%)	**0.009**	4 (3%)	5 (10%)	0.1
Septum (*n* = 168)	120 (95.2%)	48 (81.4%)		126 (93.3%)	42 (84%)	
Inferior wall (*n* = 8)	3 (2.4%)	5 (8.5%)		5 (3.7%)	3 (6%)	
The latest activation site						
Anterior wall (*n* = 4)	1 (0.8%)	3 (5.1%)	0.049	1 (0.7%)	3 (6%)	**0.01**
Lateral wall (*n* = 167)	118 (93.7%)	49 (83.1%)		127 (94.1%)	40 (80%)	
Inferior wall (*n* = 14)	7 (5.6%)	7 (11.9%)		7 (5.2%)	7 (14%)	

ESV = end-systolic volume; LV = left ventricle; NYHA = New York Heart Association. * at 12 months of follow-up.

**Table 3:** Univariate analysis of significant factors in predicting CRT response.

	Reduction * of LV ESV by ≥15%			Improvement * of at Least One NYHA Class		
	Odds Ratio	95% CI	*p*	Odds Ratio	95% CI	*p*
The earliest site in segments 2, 3, 8, or 14	**5.42**	**2.65–11.07**	**<0.001**	**3.19**	**1.57–6.48**	**0.001**
The latest site in segment 5	**3.27**	**1.56–6.89**	**0.002**	**3.23**	**1.45–7.18**	**0.004**
LVPS in segments 5, 6, 11, or 12	**10.62**	**3.71–30.43**	**<0.001**	**48.16**	**10.69–216.9**	**<0.001**
QLV interval	**1.052**	**1.029–1.075**	**<0.001**	**1.092**	**1.059–1.13**	**<0.001**
Quadripolar LV lead	**2.33**	**1.12–4.83**	**0.023**	1.69	0.81–3.54	0.2
Use of automatic CRT optimization algorithm	**2.13**	**1.07–4.22**	**0.03**	1.51	0.75–3.03	0.2
LBBB AHA	1.74	0.91–3.33	0.095	**2.18**	**1.08–4.42**	**0.03**
LBBB Strauss	2.24	0.62–8.06	0.2	**4.47**	**1.2–16.56**	**0.025**
LBBB ESC 2013	1.52	0.81–2.85	0.2	**2.35**	**1.21–4.55**	**0.011**

ESV = end-systolic volume; LV = left ventricle; NYHA = New York Heart Association; CI—confidence interval; LBBB—left bundle branch block; LVPS—left ventricle pacing site. * At 12-month follow-up.

**Table 4:** Multivariable analysis of univariate significant factors in predicting CRT response.

	Reduction * of LV ESV by ≥15%			Improvement * of at Least One NYHA Class		
	Odds Ratio	95% CI	*p*	Odds Ratio	95% CI	*p*
The earliest site in segments 2, 3, 8, or 14	**5.14**	**2.2–11.8**	**<0.001**	**3.1**	**1.2–8.1**	**0.021**
The latest site in segment 5	2.3	0.96–5.5	0.062	1.89	0.7–5.4	0.2
LVPS in segments 5, 6, 11, or 12	**4.42**	**1.37–14.3**	**0.025**	**21.2**	**4.3–105**	**<0.001**
QLV interval	**1.04**	**1.015–1.066**	**<0.001**	**1.08**	**1.04–1.1**	**<0.001**
Quadripolar LV lead	1.94	0.3–11.8	0.5	-	-	-
Use of automatic CRT optimization algorithm	1.05	0.2–5.8	0.95	-	-	-
LBBB AHA	-	-	-	1.4	0.4–4.6	0.6
LBBB Strauss	-	-	-	0.61	0.095–3.9	0.6
LBBB ESC 2013	-	-	-	0.9	0.3–3.01	0.9

ESV = end-systolic volume; LV = left ventricle; NYHA = New York Heart Association; CI—confidence interval; LBBB—left bundle branch block; LVPS—left ventricle pacing site. * At 12 months of follow-up.

## Data Availability

The raw data supporting the conclusions of this article will be made available by the authors on request.

## Abbreviations

The following abbreviations are used in this manuscript:

CRT	cardiac resynchronization therapy
ECGi	noninvasive activation mapping
LBBB	left bundle branch block
LVAT	left ventricular activation time
RVAT	right ventricular activation time
TAT	total activation time
